# The impact of operator experience on the recurrence rate for atrial fibrillation patients after radiofrequency ablation

**DOI:** 10.12669/pjms.42.7.13262

**Published:** 2026-07

**Authors:** Ping Gong, Dayong Zhang, Runfeng Zhang, Tao Zhou

**Affiliations:** 1 Ping Gong, MD, Mianyang 404 Hospital, Mianyang, No. 56 Yuejin Road, Mianyang City, 621000, China; 2 Dayong Zhang, MD, Mianyang 404 Hospital, Mianyang, No. 56 Yuejin Road, Mianyang City, 621000, China; 3 Runfeng Zhang, MD, Mianyang 404 Hospital, Mianyang, No. 56 Yuejin Road, Mianyang City, 621000, China; 4 Tao Zhou, MD, Mianyang 404 Hospital, Mianyang, No. 56 Yuejin Road, Mianyang City, 621000, China

**Keywords:** Atrial fibrillation, Radiofrequency Ablation, Recurrence, Surgeon Experience

## Abstract

**Objective:**

To explore the impact of differences in operator experience on recurrence rates after radiofrequency ablation in patients with atrial fibrillation (AF).

**Methodology:**

A total of 302 patients who underwent radiofrequency ablation of AF for the first time in Mianyang 404 Hospital from January 2019 to May 2023 were Retrospective study. They were divided into the mature operator group(study group) and the average operator group(control group). A total of 81 cases of paroxysmal AF and 88 cases of persistent AF were counted in the study group; 70 cases of paroxysmal AF and 63 cases of persistent AF were counted in the control group.

**Results:**

The cumulative ablation time and radiofrequency energy of the surgery in patients with persistent AF were less in the study group than in the control group (P<0.01). The comparison of the sinus heart rate maintenance rate at six months after the surgery in the two groups for paroxysmal AF (P>0.05) and persistent AF (P<0.05). The comparison of the sinus heart rate maintenance rate after one year of the surgery for paroxysmal AF (P<0.05) and persistent AF (P<0.05). The comparison of cumulative complications in all patients was 2.64% vs 9.27% (P<0.05).

**Conclusion:**

The level of operator experience positively correlated with the success rate of radiofrequency for AF, with fewer surgical complications in mature operators.

## INTRODUCTION

The global burden of disease study in 2019 showed that the number of patients with Afin the world was about 59.7 million, which was significantly higher than that before 2010.^1^ Previous studies have concluded that frequency control and rhythm control are equally effective for AF.^2^ However, rhythm control is now considered superior to frequency control in patients with AF with advances in medical technology and surgical instruments, The level of recommendation for catheter ablation of AF is also increasing.^3^ However, there is still a high rate of recurrence after the surgery, which is related to a variety of factors. A study showed that the maintenance rates of sinus rhythm in patients with paroxysmal AF were 67.8%, 56.3%, 47.6%, 46.6%, 35.6%, 26.5% and 30.4%, 18.0%, 3.4% respectively 5, 10, and 15 years after a single ablation.^4^ Theoretically, the more experienced the operator is, the higher the postoperative success rate is. However, there were no direct-controlled studies. Therefore, the study aimed to investigate the effect of operator experience on the recurrence rate and complications after radiofrequency ablation in patients with AF, with the hope of improving the outcome of AF and reducing the risk of the surgery.

## METHODOLOGY

The study was a retrospective study that included a total of 768 patients who underwent radiofrequency ablation of AF for the first time between January 2020 to May 2024 in Mianyang 404 Hospital . Medical record was accessed for research purposes between May 2024 to May 2025.

### Ethical approval:

This study has been approved by the Ethics Review Committee of Mianyang 404 Hospital (No.:2024-068; Date: July 31, 2024).

### Inclusion criteria:

- Patients who aged ≥ 18 years old, ≤ 80 years old for the first time to undergo radiofrequency ablation of AF.
- NYHA cardiac function class I-III.^5^
- Strictly completed a one-year follow-up (seven-day long-term Electrocardiography (ECG) in March, June, and December and ECG at any time when symptomatic).

### Exclusion criteria:

- Patients who failed to complete the expected one-year follow-up (including patients who did not undergo a strict seven-day long-term ECG).
- Patients with severe hematologic disease with contraindications to anticoagulation.
- Patients with left atrial appendage thrombus (transesophageal echocardiography or cardiac CT).
- Patients with hyper- or hypothyroidism.
- Patients with severe hepatic or renal insufficiency.

### Method section:

The patients were divided into the mature operator group (defined as having independently performed 100 paroxysmal AF surgeries and more than 50 persistent AF surgeries) (study group) and the average operator group (defined as having independently performed 30-100 paroxysmal AF surgeries and 10-50 persistent AF surgeries) (control group) according to the operator experience. After strict follow-up after operation, a total of 302 patients were counted, including 81 cases of paroxysmal AF and 88 cases of persistent AF in the mature operator group, and 70 cases of paroxysmal AF and 63 cases of persistent AF in the average operator group.

### Ablation procedure:

All patients were excluded from left atrial thrombus by transesophageal echocardiography or cardiac CT. All patients underwent general anesthesia by tracheal intubation with the cooperation of the anesthesiology department. During the operation, the femoral vein was punctured to place 10-pole electrode into the coronary sinus, with four-pole electrode placed into the apical portion of the right ventricle. Heparin was given 100 u/kg after the puncture of the interatrial septum, and activated coagulation time was monitored every hour to maintain between 250-350 s. The CARTO system (Biosense Webster, Inc., Irvine, CA, USA) + Pentaray mapping catheter (Biosense Webster, Inc., Irvine, CA, USA) was used during the operation to perform three-dimensional electroanatomic and substrate mapping modeling of the left atrium. ThermoCool SMARTTOUCH Catheter(ST) or ThermoCool SMARTTOUCH Surround Flowcatheter (STSF) (Biosense Webster, Inc., Irvine, CA, USA)was used as the ablation catheter, with the ablation parameters set as preset temperature of 43, power of 35-50 W, saline infusion of 15-30 ml/min, ablation index (AI) values of 350-380 for the posterior wall of the left atrium, 500-550 for the anterior wall, 500 for the apex, 400 for the base, and 450 for the crista. Contact pressure 5-20g.^6^ Standard circumferential pulmonary vein isolation(PVI) was performed on all patients, and the electrical isolation between the pulmonary veins and the left atrium was assessed to determine whether the surgery reached its endpoint. The patient was considered to be performed with right isthmus ablation if he/she had tricuspid isthmus-dependent atrial flutter. For patients with persistent AF, focal ablation, linear ablation, and complex fractionated atrial electrograms (CFAE) ablation be added based on PVI according to the atrial substrate. External electrical cardioversion was performed if AF was not stopped during ablation. Antiarrhythmic drugs (amiodarone or dronedarone) were used for at least three months after ablation. Data collection and follow-up: Baseline data and surgery data of patients (e.g., single-circle isolation of pulmonary vein, surgical ablation time, total radiofrequency energy and perfusion volume) were collected, and perioperative complications (e.g., venous hematoma, arteriovenous fistula, pseudoaneurysm, and cardiac tamponade) were recorded. Outpatient follow-up data were collected at three, six, and twelve months after surgery, with a seven-day long-term electrocardiogram performed in the outpatient clinic at three, six, and twelve months postoperatively, and an electrocardiogram performed at any time during the follow-up period with palpitations to look for the presence of rapid atrial arrhythmia. Any AF, atrial flutter, or atrial tachyarrhythmia episodes of ≥30 s duration recorded on routine or long-term electrocardiograms were regarded as recurrence of AF. Focal ablation: ablation of abnormal autonomic foci in atrium;Linear ablation:Connecting the top of left atrium and the bottom of posterior wall;CFAEablation:ablation of slow atrial conduction area and electrical activity disorder area^7^.

### Statistical analysis:

SPSS25.0 software was used in the study, with the measurement data expressed as mean ± standard deviation (), and the count data expressed as frequency or rate (%). The *χ2* test was used for the comparison of count data between groups and the independent samples t-test was used for the comparison of measurement data between groups, with P<0.05 indicating significant differences.

## RESULTS

### Comparison of baseline data between two groups:

In the two groups, there was no statistically significant difference between the patients with paroxysmal AF in terms of gender, age, underlying disease, left atrial size, stroke score and ejection fraction (P>0.05), and there was a statistically significant difference between the bleeding score (P<0.05); but there was no statistically significant difference between the patients with persistent AF in terms of gender, age, medical history, underlying disease, left atrial size, stroke score, bleeding score and ejection fraction (P>0.05). (Tables-I and II).

**Table-I:** Comparison of clinical data of patients with paroxysmal AF in two groups.

Item	Control group (n=70)	Study group (n=81)	χ2/t value	P value
Gender (Male/Female)	33/37	33/48	0.626	0.42
Age (*x̄*±*s*, years old)	63.50±12.41	65.23±10.05	0.934	0.35
Hypertension [case (%)]	19(27.1)	22(27.1)	0.00	0.998
Coronary heart disease [Case (%)]	20(28.5)	24(29.6)	0.02	0.887
Diabetes [Case (%)]	28(40)	31(38.2)	0.047	0.828
Left atrial size (*x̄*±*s*, mm)	34.77±5.34	34.08±4.10	-0.89	0.37
Cha2ds2-vasc score (*x̄*±*s*, score)	2.14±1.63	2.59±1.9	1.54	0.12
Hasbled (*x̄*±*s*, score)	1.04±1.08	1.58±1.22	2.83	0.01
Ejection fraction (*x̄*±*s*, %)	67.97±7.80	65.93±7.06	-1.68	0.09

**Table-II:** Comparison of clinical data of patients with persistent AF in two groups.

Item	Control group (n=70)	Study group (n=81)	χ2/t value	P value
Gender (Male/Female)	31/32	40/48	0.207	0.649
Age (*x̄*±*s*, years old)	67.30±9.78	66.18±7.61	0.759	0.45
Medical history (month)	24.30±31.33	23.96±31.89	0.064	0.94
Hypertension [case (%)]	16(25.3)	25(28.4)	0.168	0.682
Coronary heart disease [Case (%)]	19(30.1)	31(35.2)	0.426	0.514
Diabetes [Case (%)]	23(36.5)	35(39.7)	0.165	0.684
Left atrial size (*x̄*±*s*, mm)	43.44±7.25	41.68±5.01	-1.56	0.12
Cha2ds2-vasc score (*x̄*±*s*, score)	2.92±1.68	2.89±1.91	-0.076	0.94
Hasbled score(*x̄*±*s*, score)	2.00±1.35	1.77±1.38	-1.002	0.31
Ejection fraction (*x̄*±*s*, %)	62.19±8.53	59.73±10.8	-1.56	0.12

### Comparison of pulmonary vein single-circle isolation rate and ablation time in two groups of patients with paroxysmal AF:

The intraoperative immediate single-circle isolation rate of right and left pulmonary vein in patients with paroxysmal AF was higher in the study group than in the control group (P<0.05), the ablation time was significantly less in the study group than the control group (P<0.01). There were 141 supplemental ablations in the control group and 24 supplemental ablations in the study group, mainly in the top of the right pulmonary vein and the crest of the left pulmonary vein. (Tables-III and IV).

**Table-III:** Comparison of pulmonary vein single-circle isolation rate and ablation time in two groups of patients with paroxysmal AF.

Type	Control group	Study group	χ2//t value	P value
Left pulmonary vein single-circle isolation rate (%)	63(90)	77(95.06)	4.932	0.02
Right pulmonary vein single-circle isolation rate (%)	56(80)	74(91.3)	4.046	0.04
Ablation time (min)	53.27±18.65	37.50±9.89	-6.34	0.00

**Table-IV:** Distribution of pulmonary vein supplemental ablation in two groups of patients with paroxysmal AF.

Surgical supplement	Control group	Study group
Top of right pulmonary vein (%)	70(49.6)	14(58)
Right superior pulmonary vein (%)	4(2.8)	0(0)
Right inferior pulmonary vein (%)	8(5.6)	1(4.1)
Bottom of right pulmonary vein (%)	12(8.5)	1(4.1)
Top of left pulmonary vein (%)	7(4.9)	1(4.1)
Crest of left pulmonary vein crest (%)	36(25.5)	7(29.1)
Bottom of left pulmonary vein (%)	4(2.8)	0(0)
Total amount (%)	141(100)	24(100)

### Comparison of ablation time, radiofrequency energy and perfusion volume in twog roups of patients with persistent AF:

The cumulative ablation time and radiofrequency energy of the surgery in patients with persistent AF were significantly less in the study group than in the control group (P<0.01). The difference in intraoperative cold saline perfusion volume between two groups was not statistically significant (P>0.05). (Table-V).

**Table-V:** Comparison of ablation time, radiofrequency energy and perfusion volume in two groups of patients with persistent AF.

Type	Control group	Study group	t vlue	P value
Ablation time (min)	68.69±12.67	50.65±6.63	-7.233	0.01
Ablation energy (J)	95460.93±32913.93	800083.90±33423.18	-2.806	0.006
Perfusion volume (ml)	1130±580.51	976.90±510.41	-1.723	0.08

### Comparison of sinus heart rate maintenance between two groups at six months and one year postoperatively:

**Fig.1:**
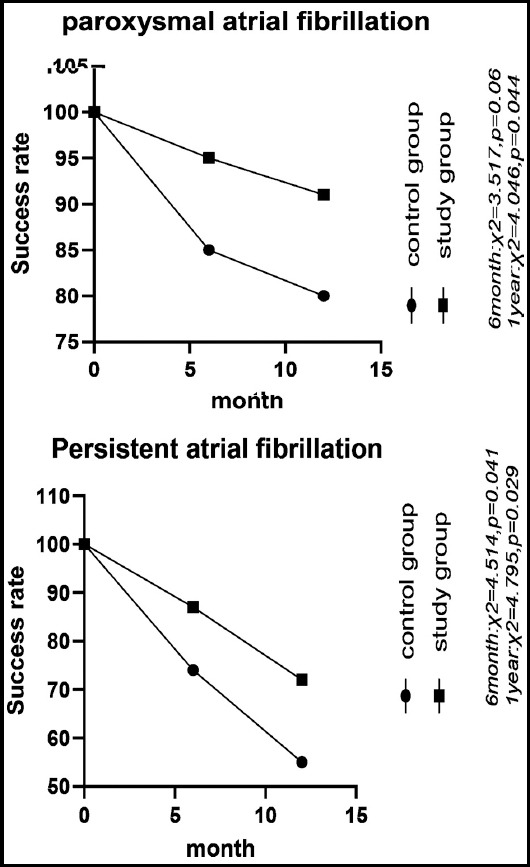
Comparison of sinus heart rate maintenance between two groups at six months and one year postoperatively.

In patients with paroxysmal AF, the comparison of cases (rate) of sinus heart rate maintenance in two groups was 60 (85.71%) vs 77 (95.06%) (P>0.05) at six months postoperatively and 56 (80%) vs 74 (91.35%) (P<0.05) at one year postoperatively. In patients with persistent AF, the comparison of cases (rate) of sinus heart rate maintenance in two groups was 47 (74.6%) vs 77 (87.5%) at six months postoperatively and 35 (55.5%) vs 64 (72.7%) at one year postoperatively, with statistically significant difference between the study group and the control group (P<0.05). (Fig.1).

### Comparison of complications between two groups:

There were 14 cases of surgical complications in the control group, including two cases of serious complications, with one case of pericardial catheter drainage and one case of emergency surgical chest opening, with statistically significant differences compared with the study group (P<0.05) (Table-VI).

**Table-VI:** Comparison of complications between two groups.

*Group*	*Venous hematoma*	*Arteriovenous fistulas*	*Pseudoaneurysm*	*Pericardial fluid (intrapericardial intubation, emergency surgical chest opening)*	*Total amount (%)*	*χ2 value*	*P-value*
Control group	4	3	4	3(1,1)	14(9.27%)	5.908	0.015
Study group	2	0	2	0	4(2.64%)		

## DISCUSSION

PVI serves as the cornerstone of all catheter-based ablation procedures for AF. In patients with persistent AF additional ablation lesions are often required alongside PVI, which is highly dependent on operator experience.^8^,^9^ This study is the first to compare procedural outcomes between paroxysmal AF and persistent AF performed by the same group of operators. The results showed that in patients with paroxysmal AF, the immediate single-circle isolation rate was superior in the study group compared with the control group, with shorter ablation times and fewer touch-up ablation points. The success rate of paroxysmal AF ablation differed between the two groups at one year but not at six months, possibly due to the routine use of antiarrhythmic drugs during the three-month blanking period and the exclusion of early recurrences within this period from the statistical analysis. A previous study by Akinori Sairaku demonstrated that experienced operators (≥50 cases/year) achieved higher one-year success rates for paroxysmal AF ablation compared with less experienced operators (<50 cases/year) (76.4% vs. 62.8%), with shorter procedure times and fewer complications, which is consistent with the findings of the present study.^10^ The criteria in our study were more stringent, requiring operators to have experience not only in paroxysmal AF radiofrequency ablation but also in persistent AF ablation, which may partly explain the higher success rates observed in both groups during follow-up. Reissmann et al. reported that even in cryoablation for paroxysmal AF, a procedure considered relatively straightforward, there were significant differences in outcomes: the first 10 cases performed by the same operator achieved a one-year freedom from AF recurrence rate of 65%, which stabilized at 85% after more than 30 cases, and operators with prior radiofrequency experience had a shorter learning curve.^11^ Although pulsed field ablation (PFA), as a novel energy source, was previously thought to create durable ablation lines without direct contact, accumulating evidence now confirms that stable contact is also essential for PFA, though its learning curve is shorter than that of radiofrequency or cryoablation.^12^ The DISRUPT-AF study showed that mean ablation time decreased from 77.92±26.85 minutes in early procedures (1st–3rd) to 62.30±26.14 minutes in later procedures (≥11th), and median fluoroscopy time decreased from 9.2 to 4.7 minutes, with all patients achieving acute PVI.^13^ However, achieving durable PVI still requires a learning curve; Kueffer et al. reported that durable PVI rates improved from 61% to 73% after 60 procedures.^14^ In patients with persistent AF, the study group had lower total ablation times and energy delivery, but there was no significant difference in intraoperative cold saline irrigation volume between the two groups. This was attributed to the control group operators’ preference for using the novel STSF pressure-sensing contact force ablation catheter. The success rates for persistent AF ablation at six months and one year were both superior in the study group compared with the control group. This difference is likely due to the different ablation strategies: all paroxysmal AF patients underwent conventional PVI alone, whereas for persistent AF, additional ablation beyond PVI was performed based on the atrial substrate. Operators with greater experience demonstrated more stable catheter manipulation and a more rational approach to deciding whether to extend ablation, recognizing that not all ablation lines achieve adequate block, even when intraprocedural verification is performed.^15^ Barbhaiya et al. showed that in a cohort of persistent AF patients undergoing their first pressure-guided ablation, the latter half of the cohort had significantly higher two-year success rates than the first half, underscoring the importance of operator experience.^16^ Another study of a novel dual-energy (PFA/radiofrequency) ablation system for persistent AF found that success rates improved significantly with operator experience: 65% in the initial phase (first five cases), 75% in the intermediate phase (cases 6–10), and 80% at one-year follow-up after more than 10 cases.^17^ The learning curve for radiofrequency ablation for AF is longer than that for cryoablation or PFA; however, due to its broad indications, flexibility in managing complex cases, and long-term reliability, it remains irreplaceable by other technologies.^18^-^20^ Radiofrequency ablation requires uniform, continuous, and transmural lesions, which depend on stable contact force and short, consecutive discharge intervals.^21^ For less experienced operators, using steerable sheaths to enhance contact force, employing deep sedation or general anesthesia to control tidal volume and reduce catheter movement, and performing regular weekly simulator training may help narrow the gap with experienced operators—a hypothesis that warrants further randomized controlled trials.

Total cumulative complications were significantly fewer in the study group than in the control group. The control group experienced 14 procedural complications, including three cases of pericardial effusion: one managed conservatively, one relieved by pericardiocentesis and drainage, and one requiring emergency surgical repair of a left atrial roof tear.

### Limitations

It is a single-center study with a small statistical sample size because of the strict follow-up requirements. In addition, the follow-up time is not long, with a seven-day long-term ECG follow-up. However, the subcutaneous electrocardiographic monitor was not placed in the follow-up, which may result in the overestimation of the success rate of the follow-up surgery. Other complications such as esophageal lesion and long-term pulmonary vein stenosis were not further explored.

### Clinical Recommendations:

1. Perform weekly simulator training, achieving a cumulative experience of at least 30 paroxysmal AF cases and 10 additional linear ablation cases before clinical procedures.
2. For regions prone to residual gaps in PVI, the first 100 cases may involve an experienced operator performing touch-up ablation.
3. Use steerable sheaths during procedures to enhance stability and improve contact force.
4. Employ deep sedation or general anesthesia to control tidal volume and reduce catheter movement.

## CONCLUSION

This study shows that the level of operator experience is positively correlated with the success rate of radiofrequency surgery for AF, with fewer surgical complications for mature operators.
